# NET-(works) in arterial and venous thrombo-occlusive diseases

**DOI:** 10.3389/fcvm.2023.1155512

**Published:** 2023-05-22

**Authors:** Monika Zdanyte, Oliver Borst, Patrick Münzer

**Affiliations:** ^1^ DFG Heisenberg Group Thrombocardiology, Eberhard Karl University Tübingen, Tübingen, Germany; ^2^ Department of Cardiology and Angiology, University Hospital Tübingen, Tübingen, Germany

**Keywords:** NET, NETosis, venous thrombembolism, atherosclerosis, vasculo-inflammation

## Abstract

Formation of Neutrophil Extracellular Traps (NETosis), accompanied by the release of extracellular decondensed chromatin and pro-inflammatory as well as pro-thrombotic factors, is a pivotal element in the development and progression of thrombo-occlusive diseases. While the process of NETosis is based on complex intracellular signalling mechanisms, it impacts a wide variety of cells including platelets, leukocytes and endothelial cells. Consequently, although initially mainly associated with venous thromboembolism, NETs also affect and mediate atherothrombosis and its acute complications in the coronary, cerebral and peripheral arterial vasculature. In this context, besides deep vein thrombosis and pulmonary embolism, NETs in atherosclerosis and especially its acute complications such as myocardial infarction and ischemic stroke gained a lot of attention in the cardiovascular research field in the last decade. Thus, since the effect of NETosis on platelets and thrombosis in general is extensively discussed in other review articles, this review focusses on the translational and clinical relevance of NETosis research in cardiovascular thrombo-occlusive diseases. Consequently, after a brief summary of the neutrophil physiology and the cellular and molecular mechanisms underlying NETosis are presented, the role of NETosis in atherosclerotic and venous thrombo-occlusive diseases in chronic and acute settings are discussed. Finally, potential prevention and treatment strategies of NET-associated thrombo-occlusive diseases are considered.

## Introduction

Diseases of the arterial and venous vascular system such as atherosclerosis, coronary artery disease (CAD), peripheral arterial occlusive disease, and venous thromboembolism (VTE) are a major health burden worldwide (1–3). Although the prevention and treatment of these diseases have been improved over the last decades, the prevalence and mortality as well as chronic complications of these cardiovascular pathologies still remain highly clinical relevant, which highlights the unmet need for novel and improved approaches in their prevention and treatment (4, 5).

Thereby, the development and progression of atherothrombosis and venous thromboembolism are highly liable to complex multicellular and multifaceted processes, known nowadays as thrombo-inflammation or immunothrombosis respectively (6, 7). Besides platelets, lymphocyte and especially neutrophil function is linked to these processes. In particular, a programmed cell death in neutrophils, designated Neutrophil Extracellular Trap formation (NETosis), was identified as a major event in both, arterial and venous thrombo-occlusive diseases. Evidence is mounting, that the NET-associated release of decondensed chromatin, pro-inflammatory as well as pro-thrombotic factors, is crucial in the formation of an atherosclerotic plaque leading to atherothrombosis, and in the pathogenesis of venous thromboembolism. Therefore, in this review article we focus on NETosis in atherosclerosis and its acute complications [e.g., acute myocardial infarction (AMI), ischemic stroke (IS)] as well as deep vein thrombosis (DVT) and pulmonary embolism (PE) which may lead to chronic complications, for instance postthrombotic syndrome and chronic thromboembolic pulmonary hypertension (CTEPH).

Thus, recent findings of NET-mediated arterial and venous thrombo-occlusive diseases are described and their common clinical implications are discussed in this review. Consequently, a brief summary on the nature of the neutrophil physiology and NETosis is followed by an overview on the role of NETosis in chronic and acute settings of arterial and venous thrombo-occlusive diseases such as atherothrombosis and venous thromboembolism. Finally, an overview of potential prevention and treatment strategies, based on modulation of NETosis or platelet function, is provided.

## Neutrophil physiology and mechanism of NETosis

### Neutrophil physiology

Neutrophils represent the most abundant subset of leukocytes and comprise 50%–70% of all white blood cells in humans. Neutrophils comprise different subpopulations, since tissue-resident, circulating, mature and immature neutrophils are described and it seems that different neutrophil subpopulations exhibit distinct characteristics and have different duties (8, 9). Nevertheless, the main characteristic features of neutrophils are their multilobulated nucleus and the numerous cytoplasmic granules containing acid hydrolases and antimicrobial peptides (10). In doing so, neutrophils are the key players in the first defense mechanism of the innate immune response and are the first immune cells arriving at the site of infection (11, 12). Upon contact with an invading pathogen in the blood stream or tissue, neutrophils are activated and release a plurality of antimicrobial peptides, enzymes and reactive oxygen species (ROS) or digest invading pathogens in a process called phagocytosis, which altogether cause the effective clearance of the pathogen. However, in general, after phagocytosis neutrophils undergo a rapid apoptosis, which is mediated by CD11b/CD18 (Mac-1) and requires production of ROS and activation of caspases (13), and are subsequently cleared from the circulation. Besides these well-acknowledged canonical neutrophil functions, a novel neutrophil mechanism of combating infections has been recently discovered. In 2004 Brinkmann and colleagues described extracellular fibers of decondensed chromatin upon neutrophil activation which were able to entrap and kill pathogens extracellularly (14). Since the first description, a growing body of evidence has revealed that NETs are of great importance in a plurality of (auto-)inflammatory and thrombo-occlusive diseases.

### The distinct pathomechanism of NETosis

Already in 1996, a novel form of neutrophil death, different to apoptosis and necrosis, was observed by Takei et al. (15), but it was only 8 years later when this lytic cell death was described in more detail and designated NETosis by Brinkmann and colleagues (14). While, in the meanwhile a non-lytic NET formation was observed as well (16–18), the lytic process of NETosis is clearly distinguishable from apoptosis or necrosis and was just recently described as well-orchestrated sequence of cellular events (19). While apoptosis is caspase-driven and associated with chromatin condensation and fragmentation, NETosis seems to be caspase-independent and is accompanied by chromatin decondensation (19, 20). In addition, unlike in necrosis, during NETosis the nuclear envelope is disintegrated, internal membranes are lost, and nuclear compounds are mixed with cytoplasmic material before plasma membrane rupture ensures the release of NETs (19, 20).

Above all, NETs are composed of expelled decondensed neutrophil chromatin, forming thread-like structures, which are approximately 15 nm in diameter (10). Subsequently, NETs provide an extracellular backbone for a plurality of neutrophil cytosolic and granular components, peptides and enzymes such as e.g., histones, myeloperoxidase (MPO), neutrophil elastase (NE) and cathepsin G (10, 19). Taken together, these extracellular chromatin meshworks create a highly pro-thrombotic and pro-inflammatory environment, which induces the interaction with endothelial cells, platelet activation and tissue damage (21, 22). In the last two decades, a multitude of infectious and noninfectious factors have been identified to induce NETosis *in vivo* and *in vitro*. NETosis can be induced by infectious pathogens such as bacteria, fungi, protozoa, viruses, as well as the bacterial cell wall components lipopolysaccharides (LPS) (23, 24), but NETs are also formed upon contact with platelets or platelet-released factors.

Platelet-leukocyte interactions depend on the platelet receptor GPIb, which interacts with the leukocyte complement receptor, Mac-1 (also known as αMβ2). Consequently, platelet membrane-expressed CD40L and soluble CD40L are known for a long time to interact with the Mac-1 receptor on neutrophils and thus increase NET formation, neutrophil adhesion and migration (25, 26). Interestingly, while under septic conditions LPS-induced platelet activation is the major driver of platelet-neutrophil interaction and NETosis, under non-infectious conditions activation-dependent CD62P expression or cleavage on the platelet surface seems to efficiently induce NETosis in mice (27, 28). In recent years, binding of platelet-derived high mobility group protein B1 (HMGB1) to the receptor for advanced glycation end products (RAGE) on neutrophils was also identified to induce NETosis and subsequent arterial and venous thrombus formation (29, 30). In addition to platelets, neutrophils are primed, and NETs exacerbate by sterile inflammation, systemic inflammatory diseases and cancer progression. Here, especially autoantibodies and immune complexes, proinflammatory cytokines and chemokines [interleukin (IL)-1β, IL-6, IL-8, tumor necrosis factor-α (TNF-α)] were shown to be able to induce NET formation (31, 32). However, employing two different intracellular activation pathways and despite the plurality of (patho-)physiological activators of NET formation, phorbol myristate acetate (PMA) and ionophores such as e.g., ionomycin and nigericin are mainly used to induce NETosis *in vitro* (33).

**Figure 1:**
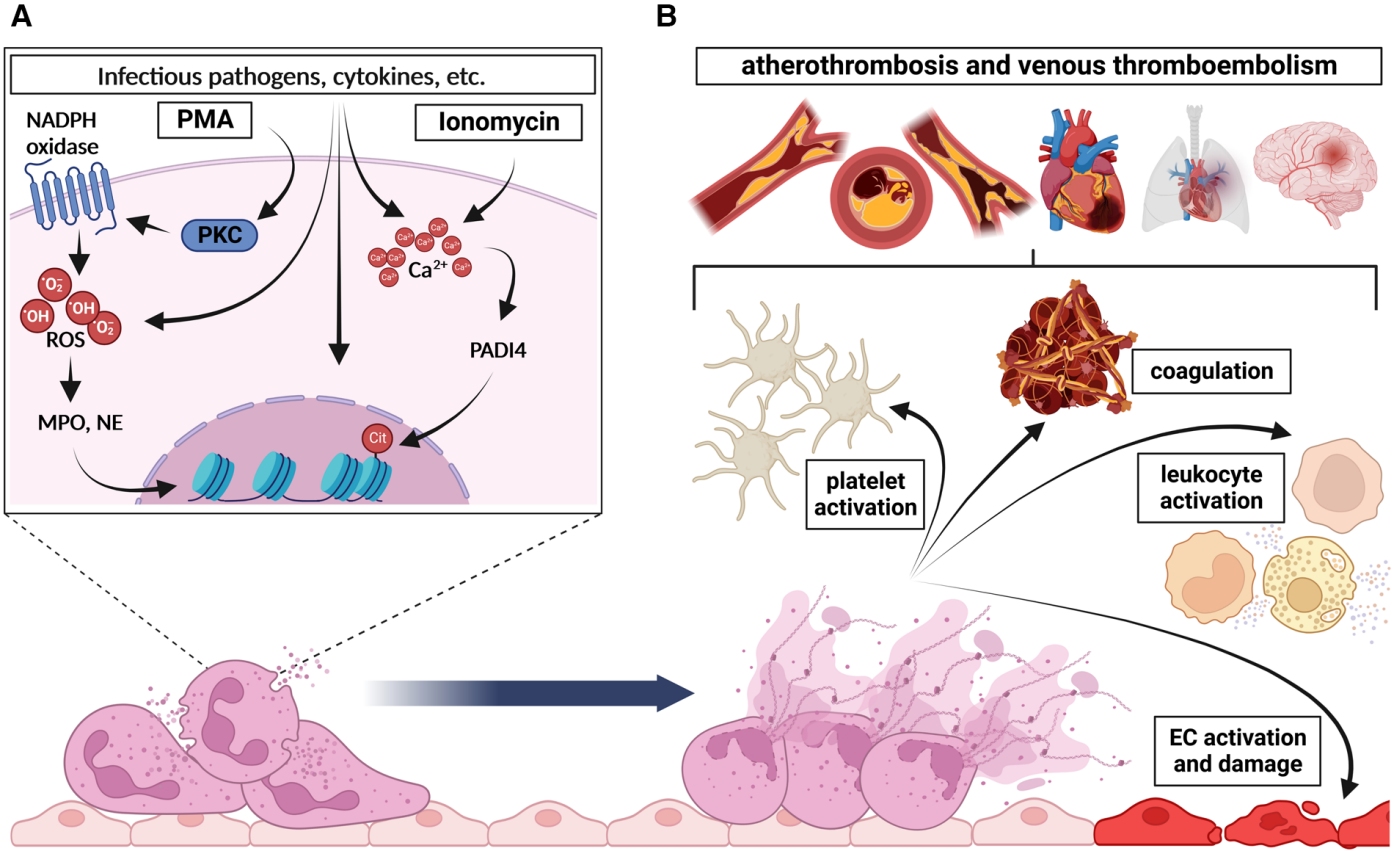
Basic mechanism of NETosis and its downstream effects. (**A**) In general, two main intracellular pathways of NETosis are distinguished: (1) Induction of NADPH oxidase by protein kinase C (PKC) upon PMA stimulation causes excessive reactive oxygen species (ROS) generation, which is followed by MPO- and NE-dependent degradation of the cytoskeleton, disruption of the nucleosome packaging, and the disintegration of the nuclear membrane, and finally leads to the release of NETs. (2) Stimulation of PADI4 by increased Ca^2+^-levels upon ionomycin stimulation for instance induces citrullination of histones, which disrupts nucleosome stability and leads to chromatin decondensation. This, together with the disintegration of the nuclear and plasma membrane, results in the release of NETs. (**B**) Once expelled in the circulation, NETs and associated molecules affect several cells and processes namely (1) platelet activation and aggregation, (2) coagulation, (3) leukocyte activation and (4) endothelial cell (EC) activation or damage respectively. Altogether, these processes finally culminate in the development and progression of atherothrombosis and venous thromboembolism. Created with BioRender.com.

The two intracellular activation mechanisms of NETosis are briefly depicted in the Figure 1 A and extensively reviewed elsewhere (8, 34). In vitro, PMA directly activates protein kinase C (PKC) due to its structural similarity to diacylgycerol (DAG) and subsequently induces the NADPH oxidase-dependent ROS generation, which leads to the translocation of MPO and NE into the nucleus (35). This translocation finally culminates in the NE-dependent degradation of the cytoskeleton, the disruption of the nucleosome packaging, and the disintegration of the nuclear membrane, leading to the release of the NETs (36, 37). However, NETosis after neutrophil stimulation with ionomycin is associated with elevated intracellular Ca^2+^ levels and relies on the post-translational deimination (citrullination) of proteins by the peptidylarginine deiminase 4 (PADI4) (38, 39). As PADI4 is the only PADI isoform able to permeate the nucleus, PADI4-mediated citrullination of histones disrupts nucleosome stability and leads to chromatin decondensation, which is a major hallmark of NET formation (38–40). Of note, PADI4-expression in neutrophils could be linked to excessive NETosis in type 1 and type 2 diabetes with subsequent impaired wound healing in mice and to an age-related increase in NETosis under resting or stimulated conditions *in vitro* (22, 41). In addition to histone citrullination, PADI4 also seems to regulate the assembly of the NLRP3 inflammasome with subsequent NETosis in neutrophils and, after secretion from neutrophils, is able to accelerate stable thrombus formation (42, 43). Both pharmacological PADI-inhibition and genetic perturbation in *Padi4*-deficient mice, resulted in significantly impaired NETosis and thrombus formation, substantiating the importance of PADI4-dependent citrullination for the NETosis process (38, 39, 44). However, neither the molecular details of ROS-dependent, nor the PADI4-induced NETosis are well understood yet, but their contribution to the interaction of neutrophils with endothelial cells and platelets or their subjection to platelet function is beyond controversy.

### Downstream effects of NETs

Due to their nature, NETs are decorated with a plurality of pro-thrombotic or pro-inflammatory molecules, proteases and reactive oxygen species. It has been proven, that NETs can further enhance platelet activation and exhibit detrimental effects on surrounding cells, thus mediating several downstream (patho-)physiological effects such as atherothrombosis and venous thromboembolism (Figure 1B).

Generally, in order to migrate in inflamed tissue, circulating neutrophils attach to activated endothelium via anti-P-selectin glycoprotein ligand-1 (PSGL-1)-mediated mechanisms (45). Consequently, a P-Selectin (CD62P)- and PSGL-1-mediated neutrophil-dependent platelet activation or platelet-dependent NET formation is conceivable (27, 46). However, as recently summarized by Kaiser and colleagues, a wide variety of platelet-neutrophil receptor pairs are known to affect their cellular functions reciprocally (47). More importantly, NETs were shown to amplify platelet function and thrombus formation more directly by presenting an intraluminal physical scaffold for platelets, propagating platelet aggregation and fostering thrombus stability (21, 48). Thereby, the effect on platelet function and aggregation was directly induced by histones, or indirectly by fibrinogen recruitment and α_IIb_β_3_-dependent or -independent mechanisms (49). Remarkably, the accepted binding of histones to toll-like receptors 2 and 4, is also known to mediate histone-induced and platelet-dependent thrombin generation (50, 51). Thereby, this histone-induced platelet activation seems to have *in vivo* relevancy as well, since intravenously injected histones caused a severe thrombocytopenia with a subsequently increased bleeding time in mice (49). In addition to histones, especially neutrophil-associated cathelicidins such as LL37 or the cathelicidin-related antimicrobial peptide (CRAMP) in mice are well-accepted inducers of GPVI-dependent platelet activation and thrombus formation (52, 53). In line with all these observations, thrombus stability was affected by NET-bound plasma protein such as von Willebrand factor (VWF), fibronectin and fibrinogen. Concurrently, NETs were also able to induce thrombin-dependent fibrin deposition, and extracellular recombinant PADI4 increased vWF-platelet string formation in mesenteric venules. Citrullination of the vWF-cleaving enzyme ADAMTS13 by PADI4 resulted in a significantly decreased enzymatic activity and thus in an impaired vWF clearance with the subsequent formation of a stable thrombus (21, 43).

Extracellular nucleic acid and its associated compounds were known for their pro-coagulant characteristic since more than a decade, but only years later the role of NET formation in coagulation was unraveled (54). Interestingly, NET-induced thrombin generation was not only platelet-dependent, but also occurred in platelet-poor plasma, where NETs were effective by activation of coagulation factors XI and XII or by impairing the thrombomodulin-dependent protein C activation (51, 55, 56). Simultaneously, together with nucleosomes the NETosis-associated proteases NE and cathepsin G inhibit the tissue factor pathway inhibitor (TFPI), and neutrophils were defined as source of factor XII that is functionally distinct from hepatic-derived factor XII (57, 58). Thus, both facts could promote a NETosis-linked pro-coagulant state in the intrinsic as well extrinsic coagulation pathway, whereas the latter is mainly induced by the presence of tissue factor (TF). The finding, that TF is enriched in NETs promote thrombotic complications in sepsis-induced lung injury, was substantiated recently by data from SARS-CoV-2 samples, in which TF-enriched NETs turned out to be key drivers of the observed severe thrombo-occlusive processes in patients (59, 60). However, NETs are not only a source of TF, but are also known to induce TF-expression in other cells.

While extracellular histones are in general toxic and NET-associated histones are known to induce epithelial and endothelial cell death, treatment of human endothelial cells with NETs increased the TF expression, thus fostering the TF activity of these endothelial cells with subsequent augmented thrombogenicity and superficial erosion (61, 62). Of note, targeted delivery of PADI4-inhibitors to experimentally eroded endothelial regions or genetically perturbated PADI4 preserved the endothelial integrity by reducing intimal NET formation (63). From this perspective, NET-associated cytokines and proteases such as interleukin (IL)-1α and cathepsin G were identified as pivotal modulators of TF-expression and thrombogenicity in endothelial cells (62). In particular, the neutrophil protease cathepsin G and cathelicidins further induce accumulation of inflammatory cells. The NET-associated release of cathelicidin-related antimicrobial peptide (CRAMP), through binding of the formyl-peptide receptor 2, mediates the recruitment and adhesion of monocytes to endothelium (64). Besides cathelicidins, cathepsin G is able to induce further accumulation of leukocytes and NETs are described to affect cytokine production on macrophages, thus contributing to inflammation progression (65, 66).

Importantly, the described characteristics and downstream effects of NETs and their involvement in platelet activation, coagulation, endothelial cell and leukocyte interaction, define NET-associated mechanisms as an ideal target in development and progression of atherothrombosis and venous thromboembolism.

## The role of NETs in atherothrombosis

### NETosis in the pathophysiology of atherosclerosis

Atherosclerosis is a lipid-driven chronic arterial thrombo-occlusive disease which is based on complex interactions between vascular and inflammatory cells as well as platelets (67). Accumulation of lipids in the endothelial cells leads to a subsequent formation of a fatty streak and an atherosclerotic plaque in the vascular wall.

Immune cells, especially neutrophils, contribute substantially to vascular inflammation and development of an atherosclerotic lesion (68). Cathelicidins (LL-37, or CRAMP in mice) were shown to induce platelet activation and secretion, which mediates platelet–leukocyte aggregate formation and neutrophil recruitment at sites of inflammation (52). Since LL-37 or CRAMP were abundantly detected in arterial thrombi in acute myocardial infarction in mice, this represents the linkage between inflammation and thrombosis in the pathogenesis of an atherosclerotic plaque (52).

There has been mounting evidence that the complex neutrophil-platelet interaction leads to NET formation, the concept called immunothrombosis, which is also a crucial counterpart in the pathophysiology of atherosclerosis (69).

Immunothrombosis represents the linkage between immune cell driven inflammation and thrombosis. On the one hand, activated neutrophils and the generated NETs participate in further activation of the coagulation cascade through the tissue factor, which is the main trigger, leading to the generation of thrombin, thrombin/PAR1-mediated platelet activation and the formation of a thrombus (70, 71). On the other hand, platelets exhibit several features, driving thrombosis: (1) platelets reinforce the coagulation cascade which leads to formation of a thrombus; (2) platelets stimulate NET formation based on the platelet-neutrophil interaction; (3) platelets secrete various proinflammatory and prothrombogenic cytokines, which directly interact with inflammatory cells.

**Figure 2:**
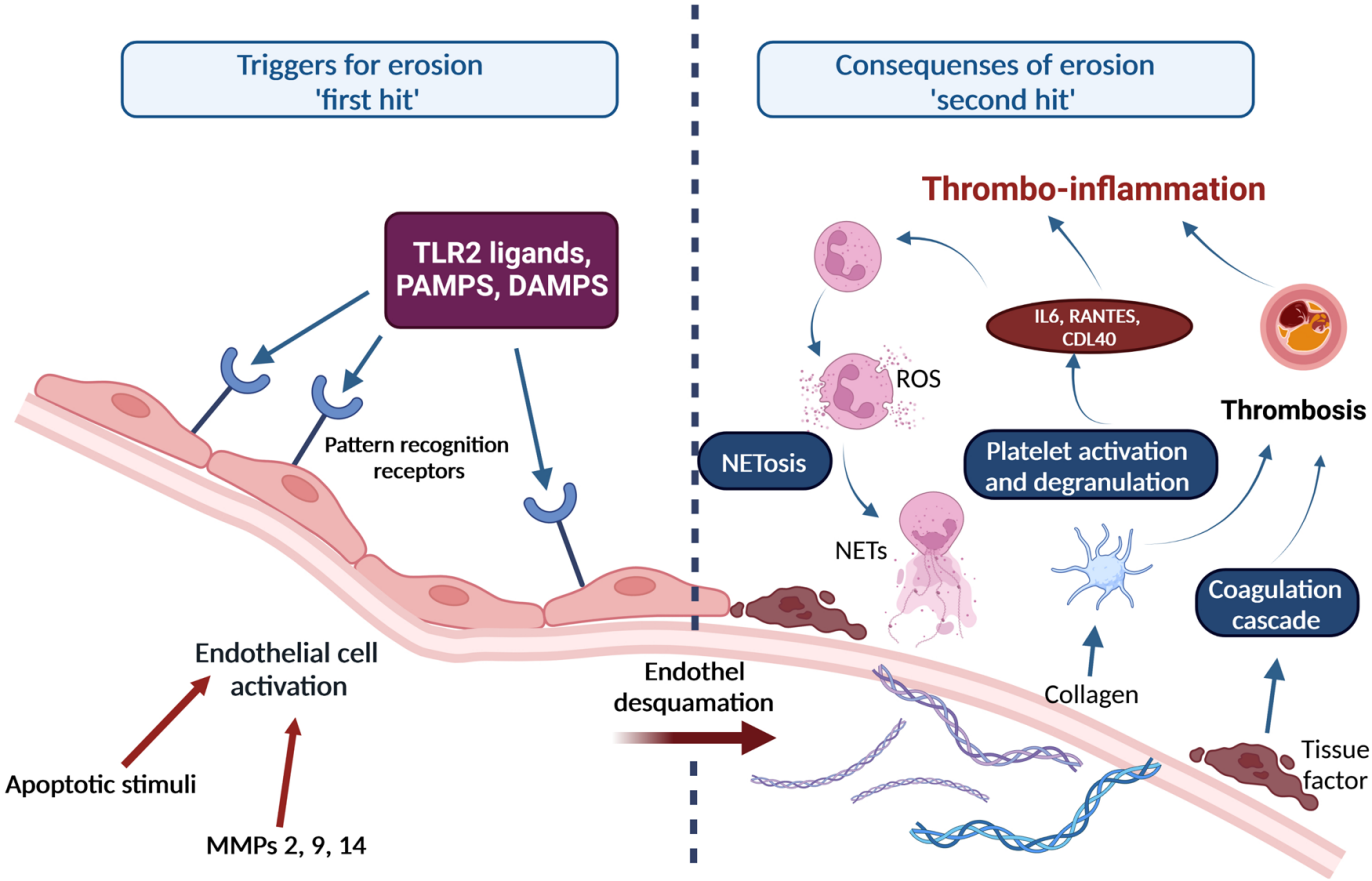
“Two-hit” theory of development of atherosclerotic lesions involving NETosis and thrombo-inflammation. Upon endothelial cell activation by MMPs or apoptotic stimuli, pathogen-associated molecular patterns (PAMPs), danger-associated molecular patterns (DAMPs) and other ligands bind to pattern recognition receptors on the surface of endothelial cells. Subsequently, inflammatory cells and modified lipoproteins promote apoptosis of endothelial cells. In parallel, matrix metalloproteinases attack constituents of the basement membrane, which leads to disruption of the endothelial cell adhesion to the basement membrane, desquamation of the endothelium, and superficial erosion (first “hit”). Several processes are initiated once the endothelial erosion starts: (1) A desquamated and dying endothelial cell releases microparticles that bear tissue factor which initiates the blood coagulation cascade; (2) Exposed sub-endothelial matrix provides a substrate for neutrophil adhesion and activation, which results in neutrophil death and formation of NETs; (3) Exposure of the sub-endothelial extracellular matrix macromolecules activates platelets causing them to degranulate and release pro-inflammatory mediators, e.g. interleukin-6 (IL-6), RANTES, plasminogen activator inhibitor-1 (PAI-1), which enhances thrombosis, activates neutrophils and further promotes NETosis, a concept named thrombo-inflammation. Adapted from Quillard et al. (64) Created with BioRender.com.

Progression of an atherosclerotic plaque may result in acute thrombo-occlusive complications, e.g., acute myocardial infarction, ischemic stroke. In summary, current research findings support a “two-hit” theory, explaining the transition of an erosion-prone chronic atherosclerotic plaque to an acute thrombosis and rupture of the plaque (Figure 2) (71, 72).

Endothelial cell damage leading to superficial erosion can be triggered by various pathogen-associated molecular patterns (PAMPs), danger-associated molecular patterns (DAMPs) and other ligands for innate immune receptors, e.g., TLR2. These ligands bind to pattern recognition receptors on the surface of the endothelial cell. Inflammatory cells abundant in atherosclerotic plaques as well as modified lipoproteins promote apoptosis of endothelial cells. In parallel, matrix degrading enzymes such as the matrix metalloproteinases attack constituents of the basement membrane, which leads to disruption of the endothelial cell adhesion to the basement membrane, desquamation of the endothelium, and, finally, superficial erosion (first “hit”) (72). Several processes are initiated once the endothelial erosion starts: (1) A desquamated and dying endothelial cell releases microparticles that bear tissue factor which initiates the blood coagulation cascade; (2) Exposed sub-endothelial matrix provides a substrate for granulocyte adhesion, activation, and degranulation, which results in the production of ROS, and neutrophil death leads to the formation of NETs; (3) Exposure of the sub-endothelial extracellular matrix macromolecules activates platelets causing them to degranulate and release pro-inflammatory mediators, e.g., interleukin-6 (IL-6), RANTES, plasminogen activator inhibitor-1 (PAI-1), which enhances the formation and stability of the thrombus, activates neutrophils and further promotes NETosis, a concept named thrombo-inflammation (72).

Finally, interaction between lipids and NETs in the pathogenesis of atherosclerosis has also been observed. Cholesterol crystals were found to be able to directly induce NETosis, as ROS production and NE translocation to the nucleus were observed in human blood-derived neutrophils (66). Inhibition of ROS production by NADPH oxidase and suppression of NE activity by proteinase 3 (PR3) proved to successfully stop NETosis and no NETs could be found in *ApoE/NE/PR3* deficient mice. Moreover, *ApoE/NE/PR3* deficient mice, substituted to an 8-week high-fat diet, exhibited a 3-fold reduction in plaque size compared to *ApoE* deficient controls, proving that NETosis is crucial in the development of atherosclerotic plaques (66). Dhawan et al. showed that the circulating level of serum extracellular DNA was significantly higher in hypercholesterolemic mice when compared to normocholesterolemic mice and the severity of hypercholesterolemia correlated positively with the levels of plasma extracellular DNA (73). Similar observations could be confirmed in patients with hypercholesterolemia (73).

Last but not least, pro-inflammatory chemokines also mediate the interplay between inflammatory cells and NETs in the pathogenesis of atherosclerosis. Interleukin-8 (IL-8) is a pro-inflammatory chemokine, which promotes inflammatory response, angiogenesis, mitosis and proliferation (74). In patients with atherosclerosis, circulating levels of IL-8 were elevated and IL-8 was found to trigger neutrophils to release NETs through the IL-8/CXCR2 signaling pathway (75). The progress of atherosclerosis was further exacerbated by activated NETs, which induced the production of IL-8, IL-6 and IL-1β from macrophages via the TLR9/NF-*κ*B pathway (75). Furthermore, administration of an anti-CXCR2 antibody effectively attenuated NET and cytokine production, and reduced the size of the atherosclerotic lesion in *ApoE^−/−^* mice subjected to high-fat diet (75).

Macrophage-derived IL-1β was shown to increase NETosis, thus antagonizing the function of IL-1β decreased the neutrophil accumulation which is associated with the extent of NETosis in atherosclerotic plaques (76).

Another pro-inflammatory cytokine, which is important in the pathogenesis of atherosclerosis is interferon α (IFN-α) due to its proven proatherogenic and pleiotropic deleterious effects on vasculature, i.e., endothelial cell damage and aberrant vascular repair, development and destabilization of atherosclerotic plaque, and enhanced thrombosis (77). The major source of IFN-α are dendritic cells. Therefore, plasmacytoid DCs which can generate various pro-inflammatory cytokines play a crucial role in development of atherosclerotic plaques. It was proved, that depletion of plasmacytoid DCs resulted in significantly decreased extent of atherosclerotic plaque formation in a mice model for atherosclerosis. In line, significantly decreased amounts of IFN-α were also observed (78). In contrast, activation of pDC by CRAMP/self-DNA complex showed significantly enhanced atherosclerotic plaque development (79). Therefore, it is possible that DNA derived from neutrophil-expelled NETs and dying plaque cells may also contribute to activation of pDCs and progression of atherosclerotic plaque (79). However, this theory needs to be further elucidated.

### NETs in the chronic setting of atherosclerosis: coronary artery (CAD), cerebrovascular (CVD) and peripheral artery disease (PAD)

In vivo findings show that NETs can be detected in atherosclerotic lesions/plaques in various vascular beds and already in early stages of the disease. NETs have been widely studied in coronary artery disease (CAD) and have been localized in coronary artery superficial erosion-prone plaques in the experimental model of coronary artery disease (80). Surrogate parameters of NETosis have also been investigated in the clinical setting. Borissoff et al. was one of the first ones to detect significantly elevated levels of extracellular DNA, nucleosomes, and MPO-DNA complexes in the circulation of patients with coronary artery disease compared to healthy controls (81). The study also observed a strong relationship between extracellular DNA, markers of NETosis (MPO-DNA complexes, citrullinated histone H4) and the presence of a prothrombotic state, determined by increased levels of thrombin-antithrombin complexes and von Willebrand Factor (vWF) (81). Therefore, surrogate parameters of NETosis may serve as a novel diagnostic marker and differentiate the acuity of CAD.

Although NETosis has been mostly investigated in coronary artery disease, recent findings show that NETs also participate in the development of atherosclerotic lesions in cerebral vessels. NETs could be found in atherosclerotic lesions of the aortic root as early as three weeks after initiation of a high-fat diet in *ApoE*-deficient mice (72, 82). NETs were localized mainly in the inner region of the carotid plaque compared to the outer region in CVD patients with carotid artery stenoses (83). Moreover, the presence of PADI4 and NETs was associated with the instability of carotid plaques (84).

NET formation in PAD has been least investigated but the findings indicate that NETosis is also of importance in the development of atherosclerotic lesions in the peripheral vascular beds. NETs have been detected in arterial thrombi obtained from patients with peripheral artery disease (85). Surrogate parameters for NETosis, circulating citrullinated histone H3 and extracellular DNA were significantly elevated in patients with PAD compared to healthy controls (86). However, further clinical and experimental studies are necessary to deepen the understanding in the role of NETosis in PAD.

### NETs in acute complications of atherosclerosis: myocardial infarction and ischemic stroke

A plaque rupture leads to an acute myocardial infarction and it has been shown that NET formation is especially pronounced in acute complications of coronary artery disease. Abundant neutrophils and NETs could be discovered in fresh and lytic coronary thrombi from patients with an acute myocardial infarction, but not in the organized thrombi (87). Moreover, a significantly increased number of neutrophil-platelet aggregates was observed in infarct-related coronary arteries from patients presenting with an acute ST-elevation myocardial infarction (71). Moreover, PMNs releasing NETs were also significantly more abundant in the blood samples and thrombi from these patients (71). Thereby, as shown in a recent work employing an experimental model of myocardial ischemia/reperfusion injury in P-selectin deficient bone marrow chimeric mice, platelet-leukocyte aggregates seem to be dispensable for myocardial ischemia/reperfusion injury as quantified by left ventricular ejection fraction (LVEF) upon 35 min of left anterior descending (LAD) coronary vessel obstruction and subsequent 24 h reperfusion (88). However, at the same time neutrophils and especially NET formation is a major driver for myocardial ischemia/reperfusion injury in mice (89). Of note, these results from experimental mouse models of myocardial ischemia/reperfusion injury have to be interpreted cautiously, since injury is induced by ligation and not an (athero-)thrombus like in humans and different times of ischemia/reperfusion are used. Thus, without additional experimental data, we can only speculate if longer time points of ischemia/reperfusion would demonstrate a P-selectin-dependent effect on myocardial ischemia/reperfusion injury for instance (88). Another study, which investigated NETosis in patients with ST-elevation myocardial infarction also found significantly more NETs in coronary thrombi compared to venous thrombi, thus showing that the role of NETosis in acute complications of coronary artery disease is indisputable (90). In line, circulating levels of surrogate parameters, that is extracellular nucleosomes and ds-DNA, were also found significantly elevated and correlated positively with the burden of NETs in coronary thrombi found in acute myocardial infarction (90).

CVD and its acute thrombo-occlusive complication, ischemic stroke, seem to be no exclusion and are also related to NETosis. Several studies showed that NETting neutrophils and NETs were found to be a common component of ischemic stroke thrombi (91–93). Since thrombi investigated in ischemic stroke patients consist of red blood cell- and platelet-rich areas, abundant amounts of extracellular DNA could be observed in the platelet-rich areas and in the boundary areas between platelet-rich and red blood cell-rich regions of the thrombi acquired from patients with ischemic stroke (94).

In line with these findings, circulating levels of NETs and extracellular DNA, as a surrogate marker for NETosis, were also found to be significantly higher in the ischemic stroke patients compared to controls (95). There has also been an observation that the thrombus morphology allows the discrimination of the stroke etiology and so neutrophils and NETs were found more abundant in presumed cardioembolic thrombi (93).

## The role of NETs in venous thromboembolism

### NETs in acute venous thromboembolic diseases: DVT and PE

Deep vein thrombosis (DVT) of the lower extremity and pulmonary embolism (PE) are the most often presentations of acute venous thromboembolism (VTE). In the US, VTE develops in an estimated 900,000 patients each year (96). Most often acquired risk factors for VTE include immobilization due to recent surgery or trauma, active malignancy, myeloproliferative disorders, central vein catheterization or transvenous pacemaker etc. Inherited thrombophilias, i.e., reductions in plasma natural anticoagulants or disturbances in downregulation of the procoagulant system and increased activity or plasma concentrations of procoagulatory factors can also result in VTE (97).

DVT has been best studied in baboon and mouse models. Venous thrombosis can occur if disturbances in venous blood flow, injury or activation of endothelium occur, or a hypercoagulable state is present. The latter are the three main features of the so-called Virchow triad. Venous stasis usually leads to hypoxemia, which triggers secretion of vWF and P-selectin from Weibel-Palade bodies in the endothelial cells and mediates platelet and leucocyte recruitment to the endothelium (98). Therefore, platelet and vWF interaction seems to be the driving force in initiating thrombosis. Accordingly, it has been shown, that vWF knockout and platelet-depleted mice are not able to form a thrombus (99).

Moreover, next to platelets and coagulation factors, neutrophils also seem to represent a key component in the initiation of a venous thrombosis (46). Experiments with mice have shown, that neutrophil-depleted mice do not develop deep vein thrombosis as well (46). Following platelet activation by TLR4, platelets bind to and activate adherent neutrophils which leads to NET formation (28). Amongst other platelet surface receptors, P-selectin is especially important in platelet-neutrophil interaction leading to NET formation in mice (27), but was negligible for platelet-induced NETosis in humans (47, 100, 101). Neutrophils also interact with coagulation system and are able to bind the factor XII and support its activation by releasing the NETs. Fuchs et al. were the first ones to induce DVT in baboons and prove the presence of NETs in venous thrombi. NETs were shown to provide a scaffold for platelets and erythrocytes and interact with fibrin fibers, which ensures the stability of the venous thrombus, whereas NET-associated histones H3 and H4 directly trigger platelet aggregation (21). Further findings showed that NET production and presence in a venous thrombus is transient. Development of a venous thrombus can be divided in stages of unorganized, organizing and organized thrombus, and accumulation of NETs was detected only during the organizing stage of the developing thrombus (102). Altogether, interactions between the innate immune system cells and coagulation system represent a novel concept of immunothrombosis.

After NET formation has been initiated, platelets seem to be able to enhance NETosis by releasing damage associated molecular pattern (DAMP) high-mobility group box 1 protein (HMGB1), which activates neutrophils and further induces NETosis (100). As already mentioned, activation of Mac-1 on neutrophils by platelet proline-rich tyrosine kinase Pyk2 and CD40L also induces NETosis (25, 103).

Inflammatory chemokines are also important elements in deep vein thrombosis and interleukin-8 is one of the best studied so far and highly associated with venous thrombosis. Ischemia induced by venous stasis leads to release of IL-8 and ROS which trigger generation of NETs (14, 20, 21). IL-8 induces integrin-based adhesion of neutrophils and monocytes to vascular endothelium and activates tissue factor on monocytes. Elevated levels of IL-8 have been found in patients with VTE. Moreover, IL-8 levels in circulation are also elevated in chronic inflammatory diseases, malignancies, and some bacterial infections, which are associated with increased risk of VTE, therefore, it is speculated, that IL-8 could be a linkage between inflammation and venous thrombosis (21).

Evidence on the role of NETosis in PE alone is currently still scarce and further studies addressing the topic are required. As already mentioned above, Savchenko et al. studied the presence of NETs in venous thrombi, which were extracted both from patients with DVT and PE. NETs could be predominantly detected in the organizing parts of the thrombus (102). Surrogate markers of NETosis, MPO, citH3 and dsDNA were found to be elevated in patients with acute VTE (104). Elevated circulating levels of citH3 were also associated with poorer prognosis of acute PE (105). However, it would be interesting e.g., to investigate the contrary role of NETs on thrombus stability and embolization in regard to acute PE and resistance of pulmonary thrombi to endogenous lytic mechanisms, but experimental data with large sample size is still lacking.

### Chronic complications of VTE: CTEPH and PTS

Recurrent DVT may result in a long-term postthrombotic syndrome (PTS), whereas an unresolved embolus of PE may lead to a chronic thromboembolic pulmonary hypertension (CTEPH).

Excessive or repetitive venous thrombosis may result in fibrotic remodeling of the vein wall, known as a postthrombotic syndrome, which is a common complication of deep vein thrombosis (106). Platelets are important contributors to postthrombotic fibrosis since they induce the recruitment of smooth muscle cells to the sight of the thrombotic injury and most probably enhance fibrosis through the transforming growth factor-β (TGF-β) signaling pathway (107). Moreover, NETs seem to be the main trigger for postthrombotic fibrosis, since they were shown to induce monocyte transformation into fibroblast-like cells which aggravated the TGF-β–mediated fibrosis (108). Subsequently, myofibroblasts release vast amounts of collagen leading to vein wall thickening, fibrosis and venous dysfunction (106).

As already mentioned above, recurrent PE may lead to unresolved pulmonary artery thrombi which may result in a chronic elevation of pulmonary arterial pressure (CTEPH). Evidence is mounting that immunothrombosis plays a crucial role in the pathogenesis of CTEPH. Markers of inflammation and NET formation, that is neutrophil counts and MPO, as well as dsDNA were found to be elevated in patients with CTEPH compared to healthy controls (108). Moreover, NET-induced upregulation of TGF-β–dependent signaling results in fibrotic remodeling of the thrombotic tissue (108).

## Clinical and therapeutic implications

The growing understanding of the pathomechanisms of NETosis and its detrimental role in a variety of vasculo-inflammatory diseases raises the questions, whether inhibiting certain steps in the process of NETosis or, on the other hand, enhancing the breakdown of NETs may be potential therapeutic approaches in order to stop or control the NET-driven diseases.

Inhibiting enzymes driving NET formation has proved to be effective and successfully reduces NET formation. For instance, one of the goals in the treatment of atherosclerosis is to stop the progression of the development of atherosclerotic plaques in arterial vascular beds. On the other hand, in case acute complications of atherosclerosis occur, e.g., acute myocardial infarction, the goal is to reduce the damage to the ischemic tissues and organs as much as possible. An overview of current findings regarding NET inhibition or degradation in cardiovascular diseases is presented in the Table 1.

**Table 1:** Summary of therapeutic approaches modulating NETosis in arterial and venous thrombo-embolic diseases.

NET-inhibiting agent	Mechanism of action		Author
Cl-amidine	PAD4 inhibitor	Carotid artery plaque, murine	Knight et al. (105)
GSK484	PAD4 inhibitor	Acute myocardial infarction, murine	Du et al. (106)
DNAse I	NET degradation	Photothrombotic stroke model, murine	Peña-Martínez et al. (107)
DNAse I	NET degradation	Atherosclerosis model, murine	Warnatsch et al. (65)
DNAse I	NET degradation	Acute myocardial infarction, human	Mangold et al. (87)
DNase I + rhADAMTS13	NET degradation	Myocardial ischemia/reperfusion (MI/R) injury, murine	Savchenko et al. (108)
DNAse I	NET degradation	Acute ischemic stroke, human	Ducroux et al. (89)
DNAse I	NET degradation	DVT, mice	von Brühl et al. (46)
DNAse I	NET degradation	DVT, mice	Brill et al. (109)
ASA	COX inhibitor	Acute lung injury, murine	Ortiz-Muñoz et al. (110)
ASA	COX inhibitor	Sepsis model, murine	Carestia et al. (111)
Clopidogrel	P2Y12 inhibitor	Acute lung injury, murine	Pulavendran et al. (112)
Clopidogrel	P2Y12 inhibitor	Renal ischemia reperfusion injury, murine	Jansen et al. (113)
Ticagrelor	P2Y12 inhibitor	Pneumonia, human	Sexton et al.(114)
Ticagrelor	P2Y12 inhibitor	Acute myocardial infarction, human	Mitsios et al. (115)

Inhibition of PADI4 has been shown to be effective in preventing the progression of atherosclerotic lesions. Knight et al. showed that pharmacological PADI4 inhibition reduces the number of NETting neutrophils, abrogates NETosis, and downregulates the interferon α (IFN-α) pathways, which results in a significantly reduced atherosclerotic lesion area and a prolonged time to thrombosis when compared to controls in a mouse model of atherosclerosis (109). PADI4 inhibition in a mouse ischemia-reperfusion model showed to reduce neutrophil infiltration, NET formation, inflammatory reaction, and cardiomyocyte apoptosis, which led to increased cardiac function improvement (110). Similar findings could be observed in the ischemic stroke mouse model, since specific inhibition of PADI4 resulted in reduction of NETosis, inhibited the formation of an arterial thrombus, and, therefore, reduced the size of the ischemic lesion (111).

The breakdown of NETs is mainly driven by DNase and its positive effects in the chronic and acute setting of atherosclerosis have been observed. Degradation of NETs by DNase I in *ApoE* knockout mice resulted in a 3-fold reduction in atherosclerotic lesion size, compared to *ApoE/NE/PR3* deficient mice, in which the atherosclerotic plaque size was unaffected by DNase I treatment (66). Application of DNase I in a mouse model of acute myocardial infarction revealed promising results. DNase activity at the infarct site correlated negatively with coronary NET burden as well as with infarct size and area at risk (90). In addition, treatment with DNAse I after an acute myocardial infarction revealed a cardioprotective effect, resulting in subsequent improvement of cardiac contractile function (89). There has also been evidence, that application of DNAse I in combination with recombinant human ADAMTS13 resulted in improved cardiac contractile function in an ischemia-reperfusion mouse model (89). DNase I also improved tissue-type plasminogen activator (t-PA)–mediated ex vivo dissolution of thrombi retrieved from ischemic stroke patients, which was shown by several studies (92, 111). This might suggest a future therapeutic approach in the treatment of acute complications of cerebral and coronary atherosclerosis.

DNAse I effects have been also widely studied in mouse models of DVT, and a significant reduction of NET production and a significantly suppressed DVT growth have been observed (46). Application of DNAse I in the model of flow restriction-induced thrombosis prevented development of venous thrombi (112).

Since platelet activation leads to platelet-neutrophil interaction and NET formation, inhibition of platelets with antithrombotic agents has also been investigated as a potential treatment strategy hindering NETosis. However, the majority of data come from the studies on acute lung or kidney injury or sepsis model. Direct platelet inhibition with acetyl salicylic acid (ASA) has been shown to reduce NET formation in a mouse model of an acute lung injury or sepsis (113, 114). It is evident that P2Y12 inhibitors, such as clopidogrel or ticagrelor, also reduce NETosis and organ damage associated with NET formation in acute lung or kidney injury (115–117). However, studies investigating whether treatment with ASA and P2Y12 inhibitors also has impact on NETosis in atherothrombotic diseases are scarce. One of them by Mitsios et al. has showed that drug eluting stents induce the generation of tissue factor bearing NETs and that ticagrelor inhibits stent-induced NET formation and therefore thrombosis (118).

The mounting evidence reveals that modulating NETosis may reverse the atherosclerotic/inflammatory changes in blood vessels, reduce the negative impact and improve the clinical outcomes of acute complications of atherosclerosis.

## Concluding remarks

Non-apoptotic neutrophil death leading to NET formation plays a crucial role in the development of atherothrombosis and venous thromboembolism and their acute as well as chronic conditions. The effect of NETs on thrombosis or vice versa are of utmost importance in thrombo-occlusive diseases. Thus, a better understanding of the pathomechanisms driving the formation of NETs may lead to the development of novel therapeutic approaches, thereby impeding the development of atherosclerosis and venous thromboembolism and/or improving the clinical outcomes of the patients suffering from its acute and/or chronic complications.
