# Supplementary material for: NET-(works) in arterial and venous thrombo-occlusive diseases
Source: Front Cardiovasc Med. 2023 May 22;10:1155512. doi: 10.3389/fcvm.2023.1155512 (PMC10239889; doi:10.3389/fcvm.2023.1155512)
Supplement: Supplementary file 1 [file Datasheet1.pdf]

**NET-inhibiting agent**	**Mechanism of action**		**Author**
Cl-amidine	PAD4 inhibitor	Carotid artery plaque, murine	*Knight et al.*[105]
GSK484	PAD4 inhibitor	Acute myocardial infarction, murine	*Du et al.*[106]
DNAse I	NET degradation	Photothrombotic stroke model, murine	*Peña-Martínez et al.*[107]
DNAse I	NET degradation	Atherosclerosis model, murine	*Warnatsch et al.*[65]
DNAse I	NET degradation	Acute myocardial infarction, human	*Mangold et al.*[87]
DNase I + rhADAMTS13	NET degradation	Myocardial ischemia/reperfusion (MI/R) injury, murine	*Savchenko et al.*[108]
DNAse I	NET degradation	Acute ischemic stroke, human	*Ducroux et al.*[89]
DNAse I	NET degradation	DVT, mice	*von Brühl et al.*[46]
DNAse I	NET degradation	DVT, mice	*Brill et al.*[109]
ASA	COX inhibitor	Acute lung injury, murine	*Ortiz-Muñoz et al.* [110]
ASA	COX inhibitor	Sepsis model, murine	*Carestia et al. 111*
Clopidogrel	P2Y12 inhibitor	Acute lung injury, murine	*Pulavendran et al. 112*
Clopidogrel	P2Y12 inhibitor	Renal ischemia reperfusion injury, murine	*Jansen et al.* [113]
Ticagrelor	P2Y12 inhibitor	Pneumonia, human	*Sexton et al.* [114]
Ticagrelor	P2Y12 inhibitor	Acute myocardial infarction, human	*Mitsios et al.* [115]
